# Mouse Abdominal Fat Depots Reduced by Butyric Acid-Producing *Leuconostoc mesenteroides*

**DOI:** 10.3390/microorganisms8081180

**Published:** 2020-08-03

**Authors:** John Jackson Yang, Minh Tan Pham, Adelia Riezka Rahim, Tsung-Hsien Chuang, Ming-Fa Hsieh, Chun-Ming Huang

**Affiliations:** 1 Department of Life Sciences, National Central University, Taoyuan 32001, Taiwan; johnjacksonyang@gmail.com; 2 Department of Biomedical Sciences and Engineering, National Central University, Taoyuan 32001, Taiwan; minhtan19906@gmail.com (M.T.P.); adeliariska07@gmail.com (A.R.R.); 3 Immunology Research Center, National Health Research Institutes, Zhunan, Miaoli County 35053, Taiwan; thchuang@nhri.edu.tw; 4 Department of Biomedical Engineering, Chung Yuan Christian University, Taoyuan 32023, Taiwan; mfhsieh@cycu.edu.tw

**Keywords:** abdominal fats, butyric acid, high-fat diet, *Leuconostoc mesenteroides*, PPAR-γ

## Abstract

The activation of peroxisome proliferator-activated rece ptor gamma (PPAR-γ) is known to induce the differentiation of adipocytes. This study aimed to investigate the probiotic effect of *Leuconostoc mesenteroides* (*L. mesenteroides*) on high-fat diet (HFD)-induced PPAR-γ activation and abdominal fat depots. Incubation of differentiated 3T3-L1 adipocytes with media of *L. mesenteroides* EH-1, a butyric acid-producing strain, significantly reduced the amounts of lipid droplets. The oral administration of *L. mesenteroides* EH-1 produced large amounts (>1 mM) of butyric acid in cecum and attenuated the HFD-induced upregulation of PPAR-γ and accumulation of abdominal fats in mice. The combination of 2% glucose with *L. mesenteroides* EH-1 increased the production of butyric acid and potentiated the probiotic activity of *L. mesenteroides* EH-1 against the formation of lipid droplets in 3T3-L1 adipocytes as well as abdominal fats in HFD-fed mice. The inhibition of free fatty acid receptor 2 (Ffar2) by its antagonist, GLPG-0974, markedly diminished the probiotic effects of *L. mesenteroides* EH-1 plus glucose on the suppression of HFD-induced PPAR-γ and abdominal fats. Besides demonstrating the probiotic value of *L. mesenteroides* EH-1, our results highlight the possible therapy targeting the butyric acid-activated Ffar2 pathway to reduce abdominal fats.

## 1. Introduction

The accumulation of fat in abdominal adipose depot is related to the development of obesity-related cardiometabolic risk, referring to the chances of having type 2 diabetes, heart disease or stroke [1]. Together, overweight and obesity are the fifth leading cause of death in the world, causing nearly 3.4 million deaths annually [2]. Genomic insights into the multiple factors that control the abdominal fat deposition have been proposed [3]. Adipocytes require several transcription factors, such as peroxisome proliferator-activated receptor gamma (PPAR-γ), CCAAT/enhancer-binding proteins (C/EBPs), signal transducers and activators of transcription (STATs) and Krüppel-like factor (KLF) proteins to facilitate the differentiation of adipocyte precursor cells into mature adipocytes [4]. PPAR-γ is a transcriptional factor belonging to the ligand-activated nuclear receptor superfamily, which directly regulates the expression of genes involved in adipocyte differentiation, lipid and carbohydrate metabolism and adipokine synthesis. Adipokine released by adipose tissues is a key factor linking obesity-induced inflammation. PPAR-γ activation induces the preadipocyte differentiation to adipocytes and stimulates triglyceride storage [5].

A high-fat diet (HFD) is largely used in animal research to induce obesity and study-related comorbidities. Although obesity results from many pathologic factors, such as genetics, unhealthy lifestyl, and eating habits, it has been proved that changes in the compositional pattern of the gut microbiota contribute in central ways to the development of various metabolic disorders. The gut microbiota regulates many host physiologic processes, including utilization of glucose, early dissociation and dihydroxylation of bile acid and biosynthesis of vitamins and amino acids [6]. Conversely, obesity further worsens the dysbiosis of gut microbiota, forming a vicious circle. Thus, the rectification of gut dysbiosis has become a promising strategy to treat obesity by drug or nutritional intervention [7]. Short-chain fatty acids (SCFAs), the main fermentation metabolites of probiotic bacteria such as butyric acid, have been shown to reduce HFD-induced metabolic disorders and inflammation [8]. Different fibres as prebiotics give rise to different amounts and patterns of the main SCFAs: Acetic, propionic and butyric acids. SCFAs are natural ligands for free fatty acid receptor 2 and 3 (Ffar2/3), found on a wide range of cell types, including adipocytes [9,10]. The various types of SCFAs with different affinities to Ffar2/3 contribute to diverse physiological effects semicolon. Ffar2, also known as G (Gi) protein-coupled receptor 43 (GPR43), is expressed in human adipose tissue and mediates the lipolytic properties of SCFAs [11]. The potency rank order of SCFAs for Ffar2 is acetic acid∼ propionic acid > butyric acid > valeric acid > formic acid [10]. Butyric acid is a vital energy source for the colonic epithelial cells and has an inhibitory effect on the growth of cancer cells in vitro [12]. Butyric acid is also thought to mediate the lipid metabolism by slowing down fat transport from the intestine [13].

*Leuconostoc mesenteroides* (*L. mesenteroides*), a heterofermentative lactic acid bacterium, is particularly well adapted to sugary niches and therefore possesses a wide spectrum of biocatalytic activities for carbohydrate modifications. *L. mesenteroides* and its enzymes have been used to produce carbohydrates and derivatives such as dextran and fructose [14]. *Leuconostoc* species are epiphytic bacteria that are commonly found in the natural environments and have been used as a probiotic bacterium for food fermentations [15]. For instance, *L. mesenteroides* starter cultures are used in some dairy and bread dough. We isolated a butyric acid-producing *L. mesenteroides* EH-1 strain from Mongolian curd cheese. Our previous study demonstrated that *L. mesenteroides* EH-1 can reduce blood glucose and increase the insulin in the model of type 1 diabetic mice induced by streptozotocin [16]. In this study, we fed mice with HFD to augment the abdominal fat depots and investigated whether Ffar2 mediates the regulation of *L. mesenteroides* EH-1 on accumulation of visceral fats. The Ffar2-mediated pathway activated by butyric acid-producing probiotic bacteria may reveal novel molecules for targeted drug therapy against visceral fat depots.

## 2. Materials and Methods

### 2.1. Ethics Statement

Institute of Cancer Research (ICR) female mice (8–9 weeks old) were purchased from the National Laboratory Animal Center, Taipei, Taiwan. Experiments were carried out in strict accordance with an approved Institutional Animal Care and Use Committee (IACUC) protocol at National Central University (NCU), Taiwan with an approved protocol (NCU-106-016, 19 December 2017). Mice were sacrificed via inhalation of CO_2_ anaesthesia. 

### 2.2. Glucose Fermentation of L. mesenteroides EH-1

For fermentation, *L. mesenteroides* EH-1 bacteria (10^7^ colony-forming unit (CFU)/mL) were incubated in rich media with the composition of 0.001% (*w/v*) of phenol red (Sigma, St. Louis, MO, USA), 1.5 g/L of KH_2_PO_4_, 10 g/L of K_2_HPO_4_, 5 g/L ofTryptic Soy Broth (TSB) (Sigma, St. Louis, MO, USA) and 10 g/L of yeast extract (Biokar Diagnostics, Beauvais, France) with or without 2% (20 g/L) of glucose. Bacteria were incubated for 24 h at 37 °C. Rich media in the absence of *L. mesenteroides* EH-1 bacteria with or without 2% glucose were included as controls. Phenol red was used as an indicator to monitor bacterial fermentation. The transformation of colour change in rich media, from red to yellow, indicating fermentation, was quantified by measuring the optical density at 562 nm (OD_562_). The reduction of OD_562_ was detected when fermentation occurred.

### 2.3. T3-L1 Cell Differentiation and Oil Red O Staining

The 3T3-L1 preadipocytes (ATCC CL-173) within 3–7 passages were cultured in Dulbecco’s Modified Eagle’s medium (DMEM) (Gibco-BRL, Grand Island, NY, USA) with 10% (v/v) foetal bovine serum (FBS) (Irvine Scientific, Santa Ana, CA, USA), 10 mmol/L of L-glutamine, 1 mmol/L of sodium pyruvate, 100 unit/mL of penicillin and 100 µg/mL of streptomycin. The differentiation of 3T3-L1 preadipocytes (0.2 × 10^5^ cells/well) was induced by treatment of post-confluent cells with differentiation medium A (DMA) consisting of DMEM, 0.5 mM of 1-methyl-3-isobutylxanthine (IBMX), 1.0 µM of dexamethasone (DEX), 1 µM of insulin (Sigma), 1% penicillin-streptomycin and 10% FBS. Each medium was then refreshed every 2 days for 10 days. Culture media (100 µL/mL) of the *L. mesenteroides* EH-1 bacteria in TSB with or without 2% glucose was added onto the culture of 3T3-L1 preadipocytes at 37 °C for 30 min on Day 0, 2, 4, 6, 8, and 10. A Whatman nylon membrane with a 0.22-µm pore size (GE Healthcare, Chicago, IL, USA) was used to filter culture media. Three wells for each treatment were conducted. The levels of lipids in 3T3-L1 preadipocytes were stained by an Oil Red O staining kit (Sigma). In brief, 4% formaldehyde fixed cells were stained with a working solution for 30 min. Lipids stained red were imaged by light microscopy and extracted in 250 µL of isopropanol for quantification via measuring absorbance at 510 using a Synergy HTX plate reader (BioTek Instruments, Winooski, VT, USA) [17].

### 2.4. Feeding Mice with L. mesenteroides EH-1 and Ffar2 Inhibition

*L. mesenteroides* EH-1 bacteria were cultured in rich medium for 24 h in 37 °C and centrifuged at 6000× *g* rpm for 5 min. The pellet was then diluted in phosphate-buffered saline (PBS). Mice were fed with 60% calorie HFD (60% fat by weight., BioLASCO Taiwan Co., Ltd.) and administered 200 µL of 2% glucose, *L. mesenteroides* EH-1 (10^7^ CFU) or *L. mesenteroides* EH-1 plus glucose by oral gavage at an interval of 3 days for 25 days. For inhibition of Ffar2, GLPG-0974 (Tocris Bioscience, Bristol, UK), a Ffar2 antagonist, was dissolved in 0.1% dimethyl sulfoxide (DMSO) to make a stock solution. GLPG-0974 (1 mg/kg body weight) was diluted in saline then was given at 1 mL/kg body weight [18] every 3 days for 25 days by oral gavage right before administration of *L. mesenteroides* EH-1 plus 2% glucose. Body weights were measured every three days. Mice were fasted for 4–8 h on the last day of treatment. The level of blood glucose from the tail blood was measured using a glucometer [16]. The abdominal fat mass was photographed and weighted. Five mice per group were used in each experiment.

### 2.5. Butyric Acid Detection by High-Performance Liquid Chromatography (HPLC)

Mouse cecum was collected after feeding mice with HFD and oral gavage of *L. mesenteroides* EH-1 in the presence or absence of 2% glucose every day for four days. Cecum was centrifuged at 5000× *g* rpm for 10 min. The supernatants were filtered through a 0.22-μm microfiltration membrane to remove bacteria and all insoluble particles. The filtrates were vortexed and equilibrated at room temperature for 5 min. Thereafter, 100 μL of concentrated HCl was added, followed by a vortex mixing step of 15 s. The samples were extracted for 20 min using 5 mL of diethyl ether. After centrifugation, the supernatant was transferred to a Pyrex extraction tube before 500 μL of a 1 mol/L solution of NaOH was added. The aqueous phase was transferred to an autosampler vial and 100 μL of concentrated HCl were added. The analysis of butyric acid was performed using an Agilent 1200 series HPLC system with a ZORBAX Eclipse XDB-C18 column (4.6 × 250 mm, 5 μm) [16]. The mobile phase consisted of 20 mmol/L NaH_2_PO_4_ solution (pH 2.2) and acetonitrile. The detector was set at 210 nm. The concentrations of butyric acid were calculated according to calibration curves of a butyric acid analytical standard.

### 2.6. PPAR-γ Expression

The 3T3-L1 cells were grown in DMEM or differentiation media for six days. Differentiated 3T3-L1 cells were treated with or without 0.1 µM of GLPG-0974 in the presence of media collected from culture of 100 µL/mL *L. mesenteroides* EH-1 bacteria (10^7^ CFU/mL) and 2% glucose for 30 min on Day 0, 2, 4, and 6. Cells were lysed with radioimmunoprecipitation assay (RIPA) buffer (Thermo Fisher Scientific). Mouse abdominal fat depots were ground and lysed with tissue lysis buffer containing protease inhibitors. Protein (30 µg) was loaded to a 10% sodium dodecyl sulphate-polyacrylamide gel electrophoresis (SDS-PAGE) gel, then transferred into a polyvinylidene difluoride (PVDF) membrane (Sigma) and blocked with 5% (*w/v*) nonfat milk before overnight incubation with primary antibody to PPAR-γ (1:1000), or β-actin (1:5000). Thereafter, the blotting was incubated with horseradish peroxidase (HRP)-conjugated secondary antibody (goat anti-mouse (1:5000) Thermo Fisher Scientific) for 1 h. Chemiluminescent detection reagent (Thermo Fisher Scientific) and Omega Lum C Imaging System (Gel Co., San Francisco, CA, USA) were used to detect protein bands, and then analyzed with ImageJ software 1.50b (National Institutes of Health, Bethesda, MD, USA).

### 2.7. Statistical Analysis 

Data analysis was performed by unpaired t-test. The *p*-values of < 0.05 (*), < 0.01 (**), and < 0.001 (***) were considered significant. The mean ± standard deviation (SD) was calculated from at least three separate experiments.

## 3. Results

### 3.1. Reduction of Lipid Droplets in Differentiated 3T3-L1 Adipocytes by L. mesenteroides EH-1

**Figure 1:**
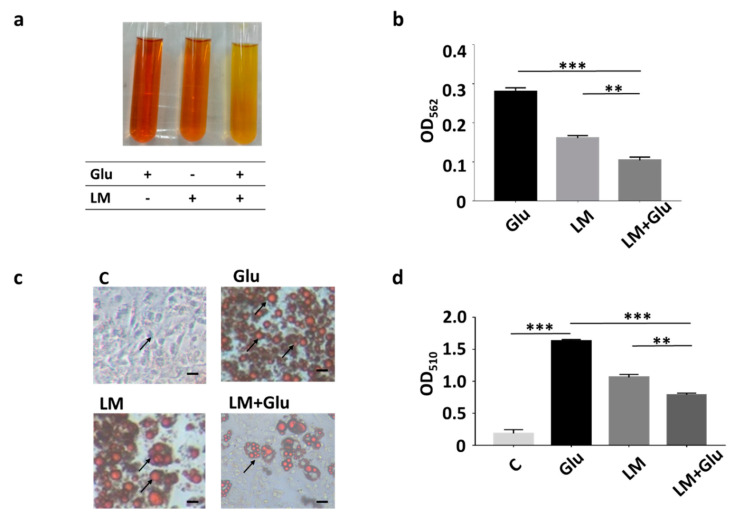
Inhibition of lipid accumulation of differentiated 3T3-L1 cells by *L. mesenteroides* EH-1. (**a**) *L. mesenteroides* (LM) was cultured in rich media with or without the addition of 2% glucose (Glu) for 12 h. Rich media added with 2% glucose alone were included as a control. Bacterial fermentation was indicated by the colour change of phenol red from red to yellow. (**b**) Media of culture with bacteria and/or glucose were measured by OD_562._ The reduction of OD_562_ was detected when fermentation happened. After 12 h, the OD_562_ value of *L. mesenteroides* (LM) with glucose (Glu) was significantly reduced (0.104 ± 0.008, *n* = 3) compared to that (0.270 ± 0.010, *n* = 3) of rich media and glucose. (**c**) 3T3-L1 cells were grown in dimethyl sulfoxide (DMEM) (**c**) or differentiation media for 10 days. Differentiated 3T3-L1 cells were incubated with media collected from culture of *L. mesenteroides* EH-1 with or without 2% glucose for 30 min. Lipid droplets (arrows) were stained by Oil Red O. (**d**) The contents of lipid droplets were quantified by measurement of OD_510_. The OD_510_ value (0.793 ± 0.021, *n* = 3) for detection of lipid droplets in differentiated 3T3-L1 cells incubated with *L. mesenteroides* (LM) with glucose (Glu) was significantly decreased compare to that (1.629 ± 0.027, *n* = 3) of cells incubated with differentiation media with glucose. Data shown represent the mean ± standard deviation (SD) of experiments performed in triplicate. ** *p* < 0.01; *** *p* < 0.001. Bar = 50 µm.

Our previous studies revealed that *L. mesenteroides* EH-1 is a butyric acid-producing bacterial strain when cultured in the presence of glucose in vitro [16]. As shown in Figure 1a,b, yellowish media and a decrease in OD_562_ were detected in the culture of *L. mesenteroides* EH-1 (10^7^ CFU/mL) for 12 h in rich media which contained 2.5% glucose in TSB (0.161 ± 0.006). Media exhibited a more yellowish colour and the OD_562_ value declined considerably upon the addition of 2% glucose into rich media of 12 h culture of *L. mesenteroides* EH-1 (0.104 ± 0.008). The data further confirmed our earlier results that glucose can trigger and enhance the fermentation of *L. mesenteroides* EH-1. We previously demonstrated that butyric acid was detectable in the culture media of *L. mesenteroides* EH-1 [16]. We next investigated whether the culture media can affect the formation of lipid droplets during the differentiation process of adipocytes. The number of lipid droplets stained by Oil Red O in 3T3-L1 preadipocytes was largely increased when DMEM was replaced with differentiation media in the cell culture, demonstrating the differentiation of 3T3-L1 cells induced by differentiation media. The incubation of differentiated 3T3-L1 cells with culture media of *L. mesenteroides* EH-1 for 30 min largely reduced the number of lipid droplets (Figure 1c,d). The addition of 2% glucose into the bacterial culture enhanced the ability of culture media to reduce the formation of lipid droplets. The result illustrated that metabolites in culture media of *L. mesenteroides* EH-1 influenced the differentiation of adipocytes.

### 3.2. In Vivo Production of Butyric Acid and Reduction of HFD-Induced Abdominal Fats by L. mesenteroides EH-1

**Figure 2:**
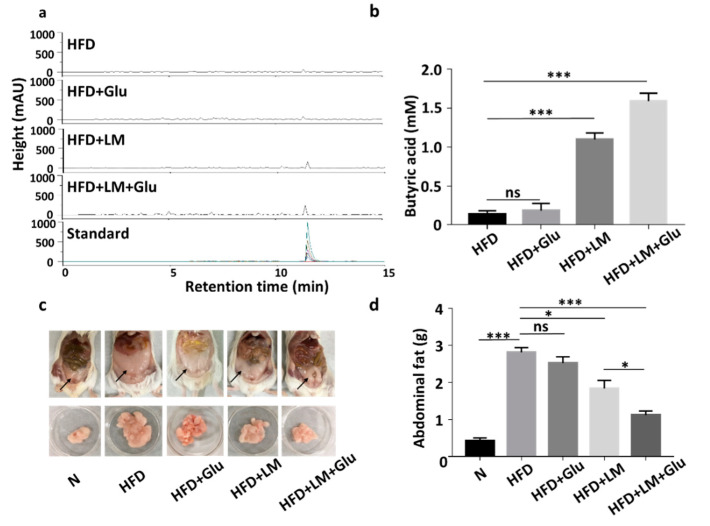
Butyric acid production of *L. mesenteroides* EH-1 in cecum and inhibition of abdominal fat depots in high-fat diet (HFD)-fed mice. (**a**) HFD-fed Institute of Cancer Research (ICR) mice were administered with *L. mesenteroides* EH-1 (LM) in the presence or absence of 2% glucose (Glu) via oral gavage every day for four days. The high-performance liquid chromatography (HPLC) chromatograms of butyric acid in mouse cecum were displayed. (**b**) The concentration (mM) of butyric acid was quantified based on the heights (milli-absorbance unit (mAU)) of standard peaks with concentrations of butyric acid from 0–20 mM. The level (1.591 ± 0.101, *n* = 5) of butyric acid in mouse cecum after LM administration was significantly increased compared to that (0.136 ± 0.044, *n* = 5) in HFD-fed mice. (**c,d**) Abdominal fat depots (arrows) in HFD-fed mice administered with *L. mesenteroides* EH-1 in the presence (1.118 ± 0.111, *n* = 5) or absence (1.838 ± 0.217, *n* = 5) 2% glucose for 25 days were dissected and weighted. Abdominal fat depots in mice fed with normal diet (N) (0.426 ± 0.072, *n* = 5) were included for comparison. Data are represented as mean ± SD of experiments performed in triplicate and five replicates. * *p* < 0.05; *** *p* < 0.001; ns = nonsignificant. Bars = 10 mm.

We next examined if *L. mesenteroides* EH-1 can produce butyric acid in vivo. HFD-fed ICR mice were given *L. mesenteroides* EH-1 via oral gavage every day for four days. Results from HPLC analysis (Figure 2a,b) showed that butyric acid concentrations of greater than 1 mM were detected in the cecum of HFD-fed mice administered with *L. mesenteroides* EH-1 (1.097 ± 0.083 mM). Administration of glucose alone did not change the level of butyric acid in cecum (0.181 ± 0.095 mM). However, oral gavage of *L. mesenteroides* EH-1 along with 2% glucose significantly enhanced the butyric acid production of *L. mesenteroides* EH-1 in the cecum of HFD-fed mice (1.591 ± 0.102 mM). The result revealed the probiotic activity of *L. mesenteroides* EH-1, which may metabolize various carbon sources in cecum to produce the substantial butyric acid *in vivo*. Furthermore, supplementation of 2% glucose in the oral gavage of *L. mesenteroides* EH-1 can promote the in vivo production of butyric acid. In parallel, we found that abdominal fat depots in HFD-fed mice (2.814 ± 0.127 g) were markedly reduced when mice were administered with *L. mesenteroides* EH-1 (1.838 ± 0.217 g) for 25 days. The reduction of HFD-induced abdominal fats by *L. mesenteroides* EH-1 was augmented when 2% glucose was included in oral gavage of *L. mesenteroides* EH-1 (1.118 ± 0.111 g) (Figure 2c,d). Moreover, supplementation of 2% glucose in the oral gavage of *L. mesenteroides* EH-1 reduced the body weight HFD-fed mice (Figure S1). The result suggested butyric acid produced by *L. mesenteroides* EH-1 in cecum may regulate the HFD-induced formation of abdominal fat depots.

### 3.3. Suppression of HFD-Induced PPAR-γ Upregulation by L. mesenteroides EH-1

**Figure 3:**
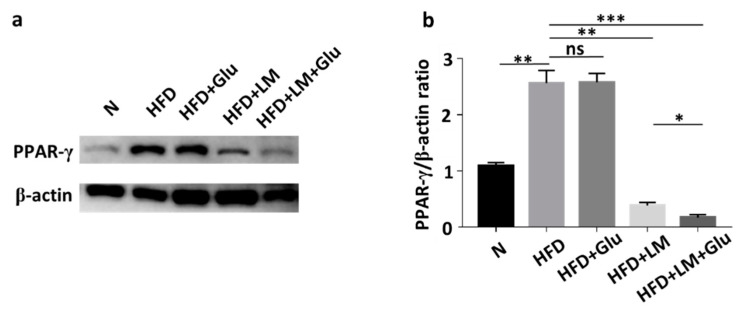
Attenuation of HFD-induced upregulation of PPAR-γ by *L. mesenteroides* EH-1. (**a**) The levels of PPAR-γ and β-actin in abdominal fat depots of mice fed with normal diet (N) or HFD along with glucose (Glu), *L. mesenteroides* EH-1 (LM) or *L. mesenteroides* EH-1 plus glucose every 3 days for 25 days were detected by western blot analysis. (**b**) The ratio intensities of PPAR-γ to β-actin were quantified. PPAR-γ expression (0.172 ± 0.050, *n* = 3) was reduced in HFD-fed mice with treatment of *L. mesenteroides* EH-1 and glucose (LM + Glu) compared to that (2.565 ± 0.224, *n* = 3) in HDF-fed mice without treatment or that (2.579 ± 0.158, *n* = 3) in HFD-fed mice with glucose. The mean ± SD for three separate experiments was calculated. * *p* < 0.05; ** *p* < 0.01; *** *p* < 0.001. ns = nonsignificant.

In agreement with the literature that HFD increased the mRNA and protein levels of PPAR-γ in mice [19], the protein level of PPAR-γ in abdominal fats was increased by two-fold when ICR mice were fed with HFD for 25 days. The upregulation of PPAR-γ by HFD was significantly suppressed in mice orally administered with *L. mesenteroides* EH-1, but not glucose alone (Figure 3). The addition of 2% glucose into the oral gavage of *L. mesenteroides* EH-1 enhanced the suppression of PPAR-γ. The result clearly illustrated the capability of butyric acid-producing *L. mesenteroides* EH-1 in abolishing the HFD-induced upregulation of PPAR-γ in abdominal fats.

### 3.4. Effects of Ffar2 Inhibitor Administration with L. mesenteroides EH-1 Probiotic Diet on PPAR-γ Level in High-Fat Diet Mice

**Figure 4:**
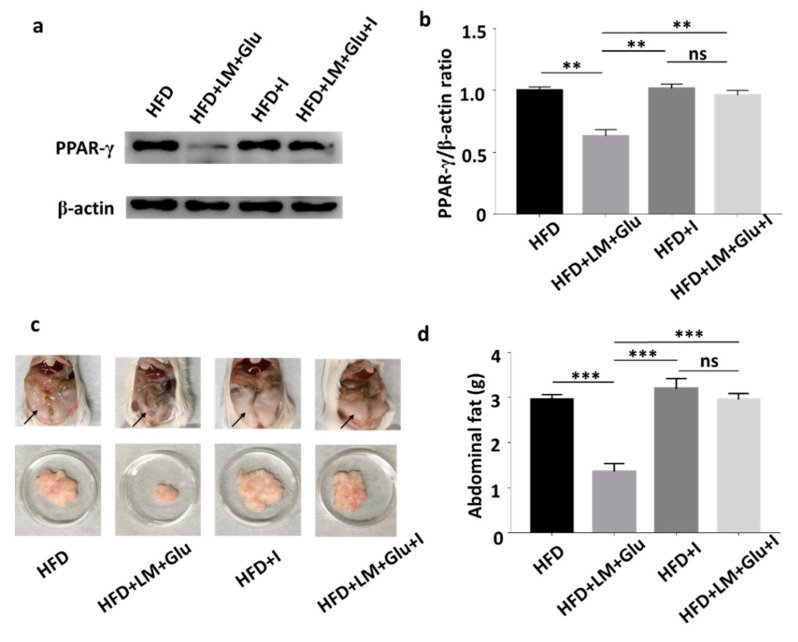
Involvement of Ffar2 in the action of *L. mesenteroides* EH-1 on the HFD-induced upregulation of PPAR-γ. HFD-fed mice were orally administered with or without *L. mesenteroides* EH-1 (LM) plus 2% glucose (Glu) right after administration of GLPG-0974, a Ffar2 antagonist (I), every 3 days for 25 days. (**a**) The levels of PPAR-γ and β-actin in abdominal fat depots were detected by western blot analysis. (**b**) The ratio intensities of PPAR-γ to β-actin were quantified. (**c**) Representative mice with abdominal fats (arrows) in the whole body or isolated abdominal fat depots were shown. (**d**) Abdominal fat depots HFD-fed mice administered with *L. mesenteroides* EH-1 with glucose in the presence (2.957 ± 0.076 g, *n* = 5) or absence (1.358 ± 0.102 g, *n* = 5) GLPG-0974 for 25 days were dissected and weighted. Data shown represent the mean ± SD of experiments performed in triplicate and five replicates. ** *p* < 0.01; *** *p* < 0.001. ns = nonsignificant. Bars = 10 mm.

As shown in Figure 2, oral administration of *L. mesenteroides* EH-1 with or without glucose can concurrently lead to the production of butyric acid in cecum and the reduction of abdominal fats in HFD-fed mice. It has been reported that butyric acid produced in the gut can enter the bloodstream and affect the physiological functions of various organs via activation of Ffar2 or Ffar3 [20]. We next examined whether Ffar2 mediates the action mechanism of butyric acid-producing *L. mesenteroides* EH-1 in suppression of HFD-induced upregulation of PPAR-γ and reduction of abdominal fats. HFD-fed mice were orally administered with GLPG-0974 to inhibit Ffar2. As shown in Figure 4, administration of GLPG-0974 alone did not alter the protein expression of PPAR-γ and the accumulation of abdominal fats in HFD-fed mice. Consistent with Figure 3, the protein expression of PPAR-γ and abdominal fats was intensely reduced when HFD-fed mice were administered with *L. mesenteroides* EH-1 plus glucose every 3 days for 25 days. However, the reduction of PPAR-γ expression and abdominal fats by *L. mesenteroides* EH-1 plus glucose were largely reversed (2.957 ± 0.076 g) when GLPG-0974 was added into the oral gavage of *L. mesenteroides* EH-1 plus glucose (Figure 4). The result demonstrated that Ffar2 was essential for *L. mesenteroides* EH-1 to attenuate the HFD-induced PPAR-γ expression in abdominal fats and formation of fat depots.

## 4. Discussion

Probiotic *L. mesenteroides* is currently used for food fermentation [21]. Here, we demonstrated, for the first time, that a butyric acid-producing *L. mesenteroides* EH-1 strain lowers the PPAR-γ expression and abdominal fat accumulation in an HFD mouse model. Our results showed that the oral gavage of *L. mesenteroides* EH-1 alone without glucose produced high amounts of butyric acid and significantly inhibited HFD-induced abdominal fats (Figure 2). The *L. mesenteroides* EH-1 may metabolize endogenous carbon sources, such as starch in the gut [22], to undergo fermentation for the production of butyric acid. Luminal glucose concentrations in the small intestine in humans are approximately 50–500 mM and exceed 300 mM after a meal. For animals on nearly physiological diets, the luminal glucose concentrations averaged 0.4–24 mM [7]. Thus, luminal glucose may be an endogenous carbon source for *L. mesenteroides* EH-1. The carbohydrates in HFD may also serve as carbon sources for fermentation by *L. mesenteroides* EH-1 before the mouse gut. After the consumption of HFD, carbohydrates may be broken down to hexose monosaccharides such as glucose, fructose and galactose [23,24] which become carbon sources for *L. mesenteroides* EH-1. It has been reported that faecal SCFAs detected in HFD-fed mice were mainly produced by gut microbiota [25].

The beneficial effects of SCFAs produced by fermentation of gut microbiota on treatments of adiposity, as well as reduction of lipid profiles, by promoting mitochondria function and energy consumption [26,27]. Body weight gain contributes to insulin-resistant glucose uptake [28]. SCFAs derived from fermentation of gut microbiota can modulate glucose homeostasis and increase insulin sensitivity [29]. However, the rapid decay of SCFAs in vivo within few hours in plasma showed the limitation of use of SCFAs as therapeutics [30]. A high concentration (greater than 1 mM) of butyric acid was detected in the cecum of mice fed with *L. mesenteroides* EH-1 with/without glucose (Figure 1b), demonstrating that *L. mesenteroides* EH-1 is a potent strain for producing butyric acid. Particularly, many reports have provided evidence that butyric acid reduced HFD-induced metabolic dysfunctions [31,32]. Studies recently reported that supplementation of butyric acid led to interrupt the PPAR-γ signaling molecules [33]. Heimann et al. showed butyric acid decreased the rate of *de novo* lipogenesis in a dose-dependent manner in rat adipocytes [34]. Our results in Figure 4 supported that activation of Ffar2 by butyric acid produced by *L. mesenteroides* EH-1 was a key event that suppressed the HFD-induced PPAR-γ expression and abdominal fats.

The expression of PPAR-γ prior to other transcription factors in adipocytes modulates the adipogenesis in exponentially growing fibroblast cell lines, demonstrating the significance of PPAR-γ in the regulation of adipocyte differentiation [35]. Consistent with other studies [35,36], our results demonstrated that differentiation media induced a significant increase in protein expression of PPAR-γ in 3T3-L1 cells. Incubation of differentiated 3T3-L1 cells with medial collected from the culture of *L. mesenteroides* EH-1 with 2% glucose downregulated the differentiation induced an increase in PPAR-γ. The treatment of differentiated 3T3-L1 cells with GLPG-0974, a Ffar2 antagonist, restored the *L. mesenteroides* EH-1 and glucose-induced downregulation of PPAR-γ (Figure S2). For in vivo experiments, HFD induced an upregulation of PPAR-γ in abdominal fats in ICR mice (Figure 3). Overexpression of Ffar2 and PPAR-γ target genes in adipose tissues was detected in HFD-fed mice [21]. This effect was associated with fat mass accumulation, increased adipocyte size and decreased basal lipolysis. It has been shown that HFD-fed Ffar2 knockout mice exhibited obesity correlated with high expressed mRNA level of PPAR-γ in abdominal fat tissues [25]. Similarly, our study demonstrated that the protein level of HFD-induced PPAR-γ in mice orally administered with *L. mesenteroides* EH-1 and GLPG-0974 was much higher than that in mice administered with *L. mesenteroides* EH-1 alone (Figure 4). Thus, the reduction of overexpressed Ffar2 may enhance the PPAR-γ-induced adipogenesis and elevate lipid accumulation [36]. In a previous study, Poirier et al. emphasized the treatment of obesity via the amelioration of PPAR-γ expression in adipocytes [37]. Our current study provided evidence that a significant decrease in PPAR-γ expression and abdominal fats in HFD-fed mice can be achieved by oral administration of probiotic *L. mesenteroides* EH-1 (Figure 3 and Figure 4).

Visceral and subcutaneous fats are two different adipose tissues that are present in the abdominal cavity in humans [38]. Subjects with visceral abdominal obesity had lower glucose disposal and oxidation as well as greater lipid oxidation when compared to those with peripheral obesity [39]. It has been documented that 20 genes, which are mostly related to lipid metabolism and glucose homeostasis, are markedly different between two types of fats. Genes encoding angiotensinogen, resistin and adiponectin in visceral fat were expressed 5-fold, 3.8-fold and 12.2-fold, respectively, higher than those in subcutaneous fat. Moreover, PPAR-γ in visceral fat is six-fold greater than that in subcutaneous fat. The visceral fat in abdominal region contains white adipose tissues, which are the fat stores associated with energetic and oxidative metabolism [40]. Moreover, Ffar2 is primarily expressed in white or abdominal adipocytes in mice compared to brown adipocytes [25]. Previous works showed that acetate and propionate inhibited isoproterenol-induced lipolysis in 3T3-L1 adipocytes [41], and the activation of Ffar2 by acetate in vivo resulted in reduced plasma levels of free fatty acids, showing the inhibition of lipolysis [42]. Our results showed that *L. mesenteroides* EH-1 can produce ample amounts of butyric acid in cecum and activate Ffar2 to mitigate the HFD-induced PPAR-γ in abdominal fat (Figure 2, Figure 3 and Figure 4). When combined with the results of previous studies, we envision that inhibition of HFD-induced PPAR-γ via activation of Ffar2 by butyric acid produced by *L. mesenteroides* EH-1 may be an effective means to reduce the abdominal fat depot.

## 5. Conclusions

The emerging contribution of intestinal microbes to the glucose homeostasis and lipid metabolism has made probiotic microbes possible for treatments of obesity [43,44]. The regulation of abdominal fat deposition via a probiotic uptake associated with lowering plasma glucose level is gaining scientific interest. In summary, we studied a probiotic strain, *L. mesenteroides* EH-1, which can produce high concentrations of butyric acid in vivo. We found that *L. mesenteroides* EH-1 activated Ffar2, downregulated PPAR-γ, and reduced abdominal fats in HFD-fed mice.

## Supplementary Materials

The following are available online at https://www.mdpi.com/2076-2607/8/8/1180/s1, Figure S1: Effect of *L. mesenteroides* EH-1 on HFD-induced weight gain in mice. Figure S2: Involvement of Ffar2 in down-regulation of PPAR-γ by *L. mesenteroides* EH-1 in the differentiated 3T3-L1 cells.
