# Supplementary material for: Mouse Abdominal Fat Depots Reduced by Butyric Acid-Producing Leuconostoc mesenteroides
Source: Microorganisms. 2020 Aug 3;8(8):1180. doi: 10.3390/microorganisms8081180 (PMC7465043; doi:10.3390/microorganisms8081180)
Supplement: Supplementary file 1 [file microorganisms-08-01180-s001.pdf]

microorganisms

MDPI

Supplementary Material

Mouse Abdominal Fat Depots Reduced by Butyric Acid-producing *Leuconostoc mesenteroides*

John Jackson Yang[1†], Minh Tan Pham[2†], Adelia Riezka Rahim[2], Tsung-Hsien Chuang[3], Ming-Fa Hsieh[4], and Chun-Ming Huang[2*]

[1] Department of Life Sciences, National Central University, Taoyuan, Taiwan.
[2] Department of Biomedical Sciences and Engineering, National Central University, Taoyuan, Taiwan.
[3] Immunology Research Center, National Health Research Institutes, Zhunan, Miaoli County, Taiwan.
[4] Department of Biomedical Engineering, Chung Yuan Christian University, Taoyuan, Taiwan.
[†]These authors contributed equally to this work.

*Correspondence: E-mial:chunming@ncu.edu.tw, Tel:+ +886-3-422-7151 x 36101

microorganisms

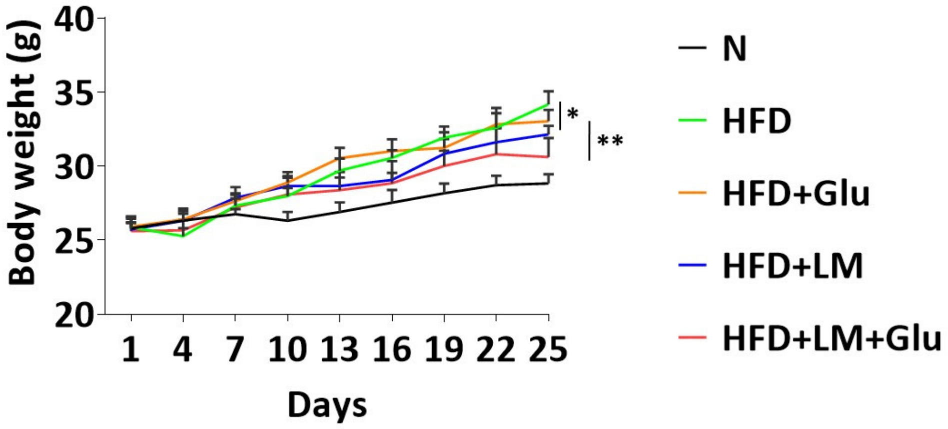

Figure S1. Effect of *L. mesenteroides* EH-1 on HFD-induced weight gain in mice. ICR mice were orally administered with normal diet (N), HFD, HFD plus 2% glucose (Glu), and HFD plus 10^7 CFU/ml *L. mesenteroides* EH-1 (LM) with or without 2% glucose. Body weights of mice were recorded every 3 days for 25 days. The mean ± SD for three separate experiments with five mice per group was calculated. *$p < 0.05$ (HFD vs HFD+LM); **$p < 0.01$ (HFD+LM vs HFD+LM+Glu).

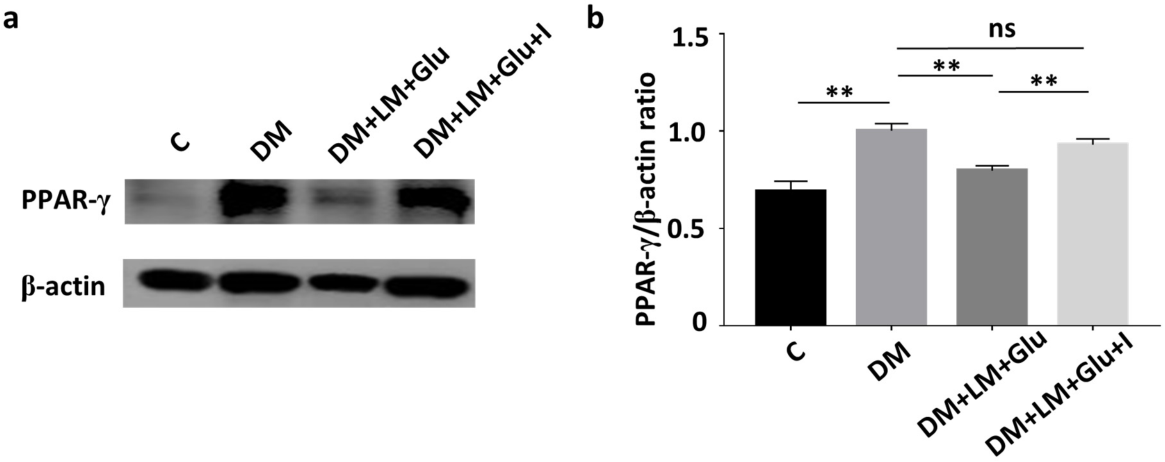

Figure S2. Involvement of Ffar2 in down-regulation of PPAR-γ by *L. mesenteroides* EH-1 in the differentiated 3T3-L1 cells. (a) 3T3-L1 cells were grown in DMEM (C) or differentiation media (DM) for 6 days. Differentiated 3T3-L1 cells treated with 0.1 μM GLPG-0974 (I), a Ffar2 antagonist, were incubated with media collected from culture of *L. mesenteroides* EH-1 (LM) with 2% glucose (Glu) for 6 days. The levels of PPAR-γ and β-actin in cells were detected by western blot analysis. (b) The ratio intensities of PPAR-γ to β-actin were quantified. The mean ± SD for three separate experiments was calculated. **$p < 0.01$. ns = non-significant.
